# Global Urinary Volatolomics with (GC×)GC-TOF-MS

**DOI:** 10.1021/acs.analchem.3c02523

**Published:** 2023-11-15

**Authors:** Antonis Myridakis, Qing Wen, Piers R. Boshier, Aaron G. Parker, Ilaria Belluomo, Evangelos Handakas, George B. Hanna

**Affiliations:** ⊥ Department of Surgery and Cancer, Imperial College London, London W12 0HS, United Kingdom; ‡ Centre for Pollution Research & Policy, Environmental Sciences, Brunel University, London UB8 3PH, United Kingdom; § Department of Urology, The First Affiliated Hospital, School of Medicine, Zhejiang University, Hangzhou 310000, China; ∥ Medical Research Council Centre for Environment and Health, School of Public Health, Imperial College London, London W12 0BZ, United Kingdom

## Abstract

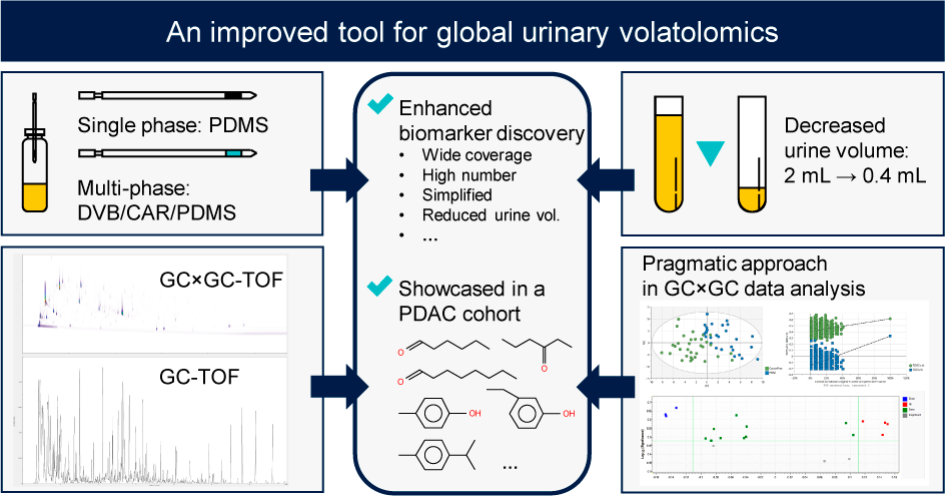

Urinary volatolomics offers a noninvasive approach for disease detection and monitoring. Herein we present an improved methodology for global volatolomic profiling. Wide coverage was achieved by utilizing a multiphase sorbent for volatile organic compound (VOC) extraction. A single, midpolar column gas chromatography (GC) assay yielded substantially higher numbers of monitored VOCs compared to our previously reported single-sorbent method. Multidimensional GC (GC×GC) enhanced further biomarker discovery while data analysis was simplified by using a tile-based approach. At the same time, the required urine volume was reduced 5-fold from 2 to 0.4 mL. The applicability of the methodology was demonstrated in a pancreatic ductal adenocarcinoma cohort where previous findings were confirmed while a series of additional VOCs with diagnostic potential were discovered.

Volatolomics is an expanding field of research with applications in disease detection and monitoring.^1^ Volatile organic compounds (VOCs), produced by cells or the microbiome, may be altered in both normal and dysregulated metabolism and as such may herald disease states. Furthermore, the detection of VOCs in different biological matrices, including exhaled breath and urine, offer the potential for noninvasive testing.^2,3^ There remains, however, a need for further optimization and standardization of current methodologies for VOCs’ detection in order to support faster uptake into to routine clinical practice.^3,4^ Furthermore, the general adoption of multidimensional chromatography–mass spectrometry (GC×GC-MS) in large scale studies still possesses a challenge. While it is undoubtedly the most powerful VOC identification technology, the issues arising from the high complexity of data alignment and analysis have not yet enabled more general applicability.^5^ Furthermore, many of the reported biomarkers are also seldom validated in follow-up studies.^4^

We had previously developed a pipeline for urinary volatolomics focusing on the nonpolar fraction of the volatolome and by employing single-dimension gas chromatography online with time-of-flight mass spectrometry (GC-TOF-MS).^6^ Herein, we report an updated methodology for urinary VOC analysis, where we (i) achieve a global coverage of the volatolome by using multiple sorbent materials with varied polarities, (ii) increase throughput by consolidating our previous two single-dimension GC methods to a unified one, (iii) minimize urine sample consumption 5-fold (from 2 to 0.4 mL), and (iv) employ GC×GC-MS in combination with tile-based analysis which aids the discovery of more VOCs with diagnostic value.

## Experimental Section

### Chemicals and Consumables

Analytical grade hexane, methanol, sodium chloride, hydrochloride acid (HCl, 37% v/v), *n*-alkane mix (n-C_8_ to n-C_20_ in hexane, 40 mg/L), and isotopically labeled analytical standards, including acetone-d_6_, acetophenone-d_8_, benzene-d_6_, butyraldehyde-d_2_, phenol-d_6_, and toluene-d_8_, were purchased from Sigma-Aldrich (Gillingham, UK). Nanopure water was produced by a Millipore Direct-Q 3 water purification system (Merck Millipore, Watford, UK). Cryovials and centrifuge tubes were provided by Scientific Laboratory Supplies Ltd. (Nottingham, UK). Crimp-top, amber glass, 20 mL headspace vials, caps with sorptive extraction septa, HiSorb Agitator, single-phase (polydimethylsiloxane (PDMS) coated HiSorb, p/n: H1-AXAAC) and multiphase (divinylbenzene (DVB)/Carboxen (CAR)/PDMS HiSorb, p/n: H4-AXAAC) sorptive extraction probes, stainless steel thermal desorption (TD) tubes (Biomonitoring; Carbograph/Tenax sorbents, p/n: C2-AAXX-5149), and DiffLok TD caps were provided from Markes International (Llantrisant, UK).

### Urine Samples

Urine samples from five healthy volunteers were collected for the purpose of development and optimization (REC reference 04/Q0403/119). Subjects were asked to provide a first morning urine sample in a standard 50 mL urine specimen vial. No specific dietary restrictions were requested prior to sampling. Nanopure water (18.2 MΩ) was collected in the same vials to evaluate blank contamination levels. All samples (urine, pooled urine, water, and dilution series of 1%, 2.5%, 5%, 10%, 20%, 40%, 60%, and 80% pooled urine to water) were transferred to 2 mL conical bottom, polypropylene tubes in 0.4 mL aliquots. Samples were spiked with 10 μL of an isotopically labeled internal standard mixture (acetone-d_6_, acetophenone-d_8_, benzene-d_6_, butyraldehyde-d_2_, phenol-d_6_, and toluene-d_8_, 2 mg/L in MeOH-H_2_O 1:1) and stored at −80 °C until analysis.

Clinical applicability was assessed by analyzing the urine of 28 pancreatic ductal adenocarcinoma (PDAC) patients and 33 cancer-free control patients with benign pancreatic pathologies (REC reference 17/WA/016 and 14/LO/1136). Cohort details are described elsewhere.^6^ Briefly, the inclusion criteria were as follows: (i) adult PDAC patients and (ii) adult control patients with normal a upper gastrointestinal tract on computed tomography (CT). The exclusion criteria were as follows: (i) presence of other types of pancreatic or synchronous cancers, (ii) benign gastrointestinal conditions, and (iii) presence of active infection, liver failure, or renal failure. Samples (0.4 mL aliquots) were analyzed in three analytical batches, including five QC and one blank sample in each batch. A dilution series was analyzed as well before the clinical sample analysis.

Urine containers were filled to their maximum capacity to minimize headspace generation, were temporarily stored at 4 °C right after collection, and were aliquoted and frozen at −80 °C within 8 h. Containers were constantly kept capped, and aliquoting was performed in dry ice.

### VOC Extraction

All sample handling was performed in dry ice except during headspace generation. Vials and tubes were kept constantly capped to minimize VOC losses. HiSorb probes, which were used for urine extraction, consist of sorbent(s) stabilized in inert-coated, stainless steel probes which are inserted through septa to headspace vials and subsequently inserted onto stainless steel tubes and analyzed with thermal desorption (TD). TD tubes and sorptive extraction probes were conditioned before use according to the manufacturer’s instructions on a TC-20 tube conditioner (Markes International, UK, p/n: R-TC20-2) for 2 h at 310 and 280 °C, respectively, under N_2_ (99.9995%) flow at 50 mL/min. Urine samples were thawed at 4 °C and then transferred with a 5 mL pipet to 20 mL headspace vials with 1.6 mL of buffer (1% HCl saturated aqueous NaCl solution) added. The pH of the buffered samples was 2, and the generated headspace was at 18 mL.

For sorptive extraction analysis, probes were either inserted into the headspace or immersed in the liquid phase. Samples were agitated at 300 rpm and 60 °C for 1 h using a HiSorb agitator. Finally, probes were transferred to their corresponding empty TD tubes.

### TD-(GC×)GC-MS Analysis

All analyses were carried out with a TD-(GC×)GC-TOF-MS system from SepSolve (Peterborough, UK) on the same day as the sample extraction. Thermal desorption was performed with a Markes International TD-100-xr system, gas chromatography with an Agilent 7890B GC instrument equipped with a SepSolve reverse-fill, flush, INSIGHT flow modulator, and mass spectrometry with a Markes International BenchTOF-Select instrument. Biomonitoring TD tubes and multiphase and single-phase sorptive extraction probes were initially prepurged for 1 min with He flow at 50 mL/min. Primary desorption was performed at 260 °C/15 min for sorptive extraction probes and at 280 °C/8 min for TD tubes, and VOCs were directed onto a focusing trap (Material Emissions, Markes International) at 25 °C in splitless mode. Trap (secondary) desorption was common for GC and GC×GC analysis and performed at 300 °C (ballistic heating at 100 °C/s) for 3 min, with the flow path onto the GC instrument heated constantly at 200 °C. During focusing trap desorption, split ratios of 5.3:1 and 5:1 were used for GC and GC×GC, respectively. The focusing trap desorption split flow was recollected onto conditioned Biomonitoring TD tubes. GC analysis was performed on an Rxi-624Sil MS column (30 m × 0.25 mm × 1.40 μm, Restek, Saunderton, UK). He flow was set at 1.4 mL/min, constant flow. The oven temperature was initially held at 40 °C for 1 min, increased to 280 °C at a rate of 10 °C/min, and finally held at 280 °C for 10 min. GC×GC analysis was performed on a primary WAX-HT column (20 m × 0.18 mm × 1.4 μm, MEGA S.r.l., Legnano, Italy) and a secondary VF200ms column (5 m × 0.25 mm × 0.10 μm, Agilent Technologies, Santa Clara, USA). Primary column flow was set at 0.5 mL/min and secondary column flow at 20 mL/min with He as the carrier gas. The oven temperature was initially held at 50 °C for 3 min, increased to 260 °C at a rate of 10 °C/min, and finally held at 260 °C for 10 min with a modulation period of 4 s and a flush time of 100 ms.

### Data Extraction, Preprocessing, and Statistical Analysis

(GC×)GC-MS data were aligned and baseline corrected with dynamic baseline compensation (peak width: 6 s for GC; 0.2 s for GC×GC) with ChromSpace (Markes International). Dynamic baseline compensation is a proprietary background correction algorithm which selectively eliminates ions resulting from chromatographic background noise (e.g., column bleed). It takes the user-defined average peak width, multiplies this number by 10, and then checks the entire datafile for any ions that remain constant during this time window. Any ions that remain constant are eliminated, thereby creating a cleaner baseline for improved detection and identification of trace peaks. Structural annotation was performed with the NIST 20 Mass Spectral and Retention Index Libraries (NIST, Gaithersburg, USA) and Wiley Registry of Mass Spectral Data, 8th edition (Wiley, New Jersey, USA) mass spectral libraries and was facilitated by analyzing all the pooled QCs recollected in a single tube to boost sensitivity in low abundance VOCs. GC×GC data were analyzed with a tile-based fisher ratio approach^5^ with ChromCompare+ (Markes International, Llantrisant, UK). Features with <50% presence in the data set or with abundance < 1000 counts were removed. The data set was normalized with probabilistic quotient normalization and log10 transformed.^5^ Features with T-statistic > 3.0 and fold change > 1.08 were considered relevant for group separation. For GC-MS data, peak deconvolution was performed with ChromSpace (Markes International, Llantrisant, UK) and peak integration with Gavin.^7^ Siloxanes (artifacts generated either from chromatographic columns or extraction sorbents), features with signal-to-noise ratio (S/N) < 3, and annotated features whose reverse matched factor (RMF) < 800 were not further analyzed. A pooled quality control (QC) approach, which has been described elsewhere, was applied where preprocessing steps were evaluated with principal component analysis (PCA).^6^ Briefly, features with either (i) CV > 30% in the pooled QC samples or (ii) blank average levels < 30% in nanopure water compared to their corresponding levels in the pooled QC or (iii) 1-tailed Spearman’s rho > 0.7 in the dilution series and q value < 0.05 after Benjamini–Hochberg correction were removed from further analysis. Multiple comparison correction was performed in MATLAB (MathWorks, Natick, USA).^8^ Orthogonal partial least-squares discriminant analysis (OPLS-DA) and PCA were performed with SIMCA 17 (Sartorius, Malmö, Sweden). The variable importance projection (VIP) score was used to select the features with the highest discrimination potential;^9^ VIP-score > 1.35 was considered relevant for group separation. Permutation testing and CV-ANOVA were used to validate the OPLS-DA model.

## Results and Discussion

### Optimization of VOC Extraction and GC-MS Analysis

**Figure 1:**
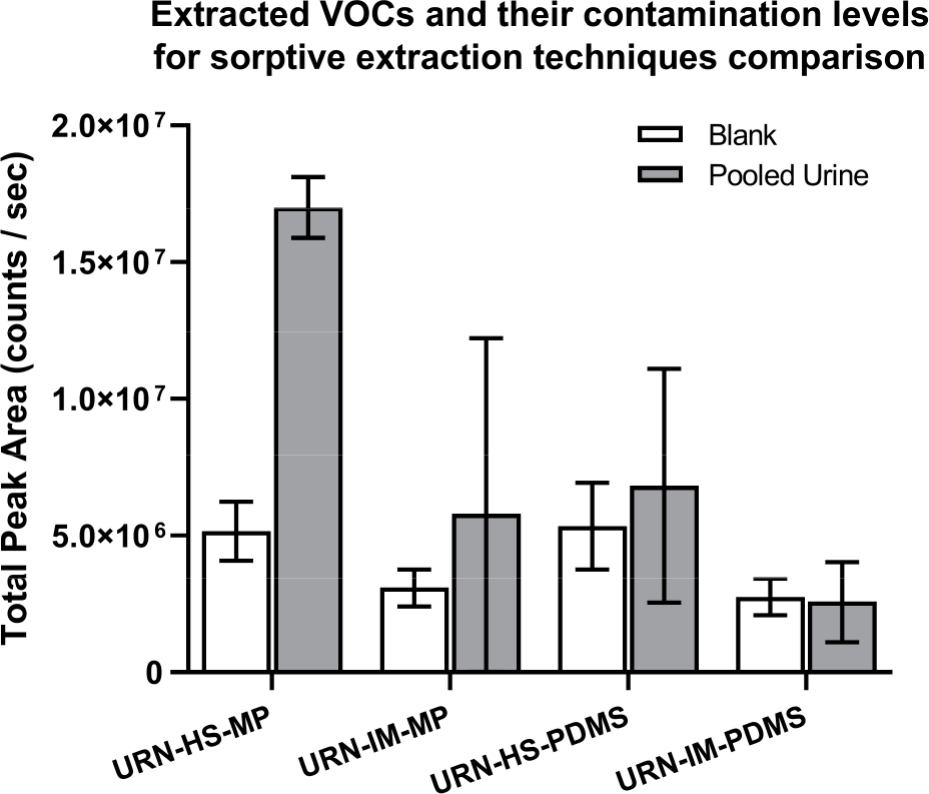
Extracted VOCs and their contamination levels for comparison of sorptive extraction techniques, where the multiphase sorbent approach in headspace analysis clearly outperforms all the rest in terms of total peak area of summed extracted VOCs: URN, urine; HS, headspace; IM, immersive; MP, multiphase; PDMS, polydimethylsiloxane.

Multiphase sorbent (PDMS/CAR/DVB) and single-phase sorbent (PDMS) sorptive extraction probes were evaluated, both in immersive and headspace sampling modes. Both methods were chosen for their potential to be adapted for high throughput and, therefore, applicability for large scale clinical studies. A pooled healthy volunteer urine sample and corresponding blank (nanopure water instead of urine) were analyzed with GC-TOF-MS in replicate (5 pooled urine + 5 blank samples/extraction condition). A series of VOCs were identified and integrated with the use of an in-house RT/mass spectra library. Peak areas (counts/s) were summed for all integrated VOCs, and the five replicates were averaged and are presented in Figure 1. The error bars represent the standard deviation between the five replicates in each examined condition. Multiphase/headspace sorptive extraction outperformed other methods in terms of both recovery and reproducibility, with the contamination being similar in all four conditions (multiphase or single-phase sorptive extraction in immersive or headspace sampling, Figure 1). Compared to single-phase sorptive extraction, the total recovery was more than doubled for multiphase sorptive extraction with substantially better reproducibility.

While the addition of HCl might compromise the recovery of basic compounds, short chain fatty acid imbalances have been associated with numerous health conditions and particularly with pancreatic ductal adenocarcinoma.^10^ The effect of HCl addition and extraction under similar conditions has been studied extensively in our previously published work. We showed there that multiple VOC categories of key chemical classes were benefited by extraction under the same conditions with the present study and that there was no evidence of artifact formation.^6^ Also, it should be noted that sorptive extraction is less prone than traditional headspace, since the VOCs are continuously trapped onto the sorbent material and become less reactive.

Due to the diversity in physicochemical properties of the urinary VOCs and with the aim to develop a global methodology, a chromatographic column with a midpolar phase (Rxi-624Sil) was selected for our GC-TOF-MS assay and an analytical run time of 35 min was developed. This method outperformed our previously published methodologies (167 and 121 VOCs for nonpolar and polar assays, respectively^6^) by measuring 195 VOCs which passed the same set of QC criteria in terms of linearity, reproducibility, and contamination. It is worth mentioning that sample volume was decreased 5-fold compared to the previously described assays (0.4 vs 2 mL). Therefore, a methodology is presented which detects a higher number of reliably measured VOCs and the same time reduces the consumption of valuable patient samples and increases throughput (57 + 44 = 101 min for polar + nonpolar assays vs 35 min in the current unified method) by achieving this performance in a single chromatographic run. This was accomplished by the incorporation of the multiphase sorbent sorptive extraction, the use of a universal column phase which enabled the reliable analysis of a wide polarity range of VOCs, and finally, the optimization of data preprocessing.

It is worth noting that multiphase sorbent sorptive extraction probes are more expensive and have a shorter life span compared to single sorbent PDMS-based ones, a challenge that we hope to overcome in the future.

**Figure 2:**
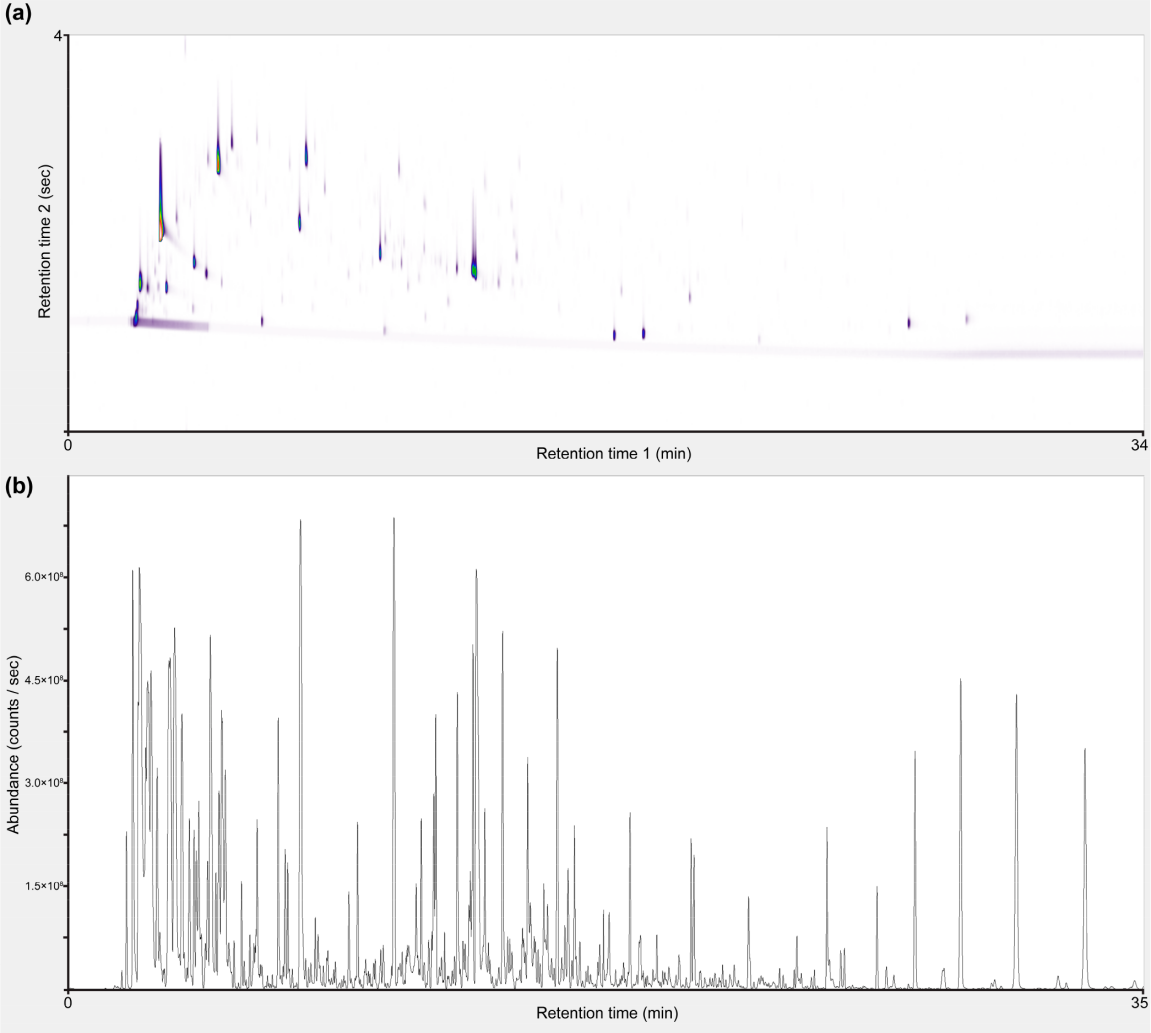
GC×GC- (a) and GC- (b) TOF-MS chromatograms of recollected pooled quality control (QC) urine samples. The recollection of multiple samples to a single thermal desorption (TD) tube facilitated metabolite annotation.

Furthermore, to enhance VOC annotation, all 21 pooled QC samples were recollected in a single tube and were analyzed with both GC- (Figure 2a) and GC×GC-TOF-MS (Figure 2b). With this approach, the bottleneck of all pooled QC strategies was effectively tackled where not all features are detectable in the pooled mix samples. Features which are at concentrations close to the detection limits and not present in all study samples often cannot be detected in the pooled QC, since their average levels in the pooled QC samples fall below detection limits. However, with our recollection approach, the concentration levels on the recollected single tube are substantially higher (sum instead of average levels), which facilitates effective detection and identification of more features.

### Urinary VOCs of Pancreatic Ductal Adenocarcinoma (PDAC)

**Figure 3:**
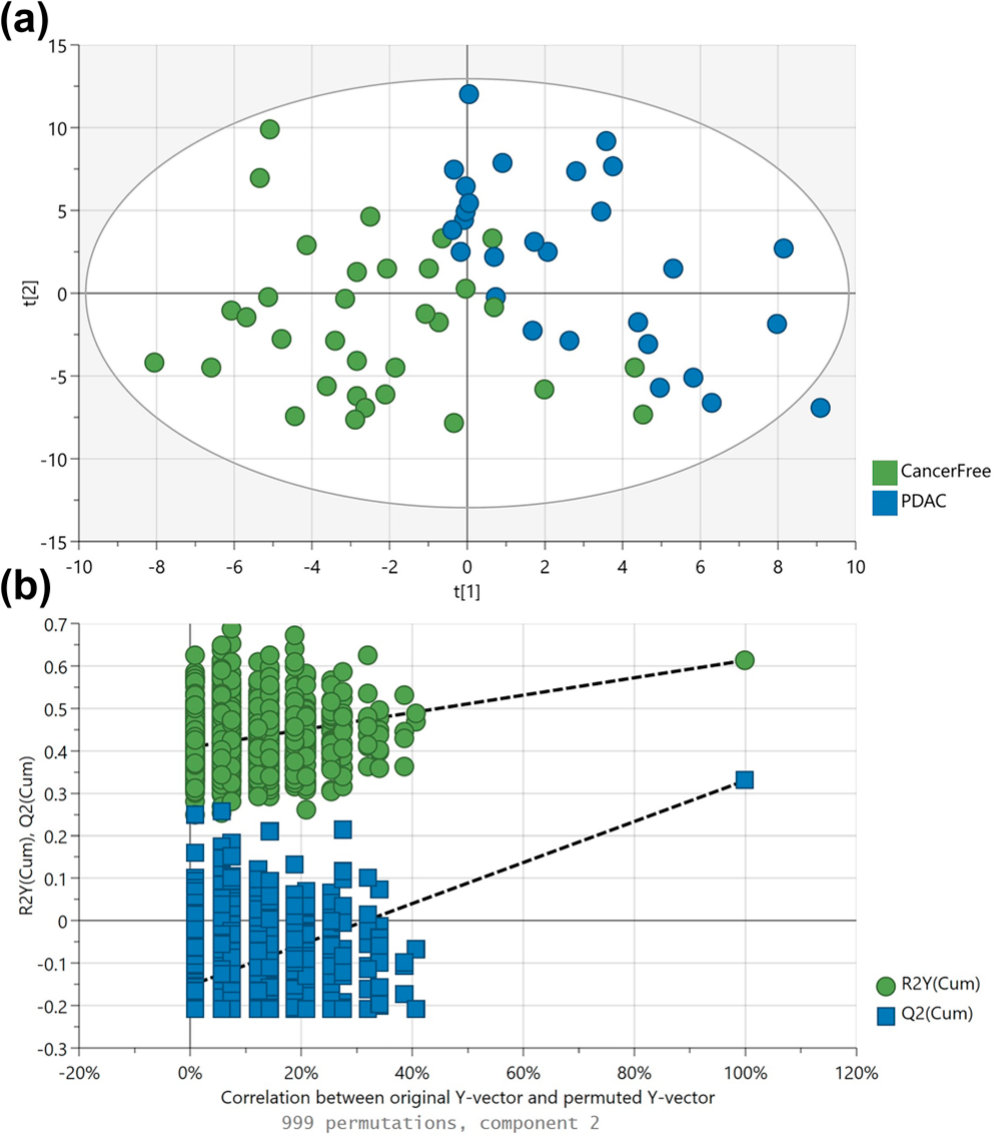
(a) Score plot of the OPLS-DA model generated on the preprocessed GC-TOF-MS data set, indicating cancer-control separation. (b) Permutation testing – 999 repeats: The *Y*-axis represents the R^2^Y and Q^2^ values of each model. The *X*-axis shows the correlation coefficient between the “real” Y and the permuted Y. R^2^Y, 0.61; Q^2^, 0.32.

**Figure 4:**
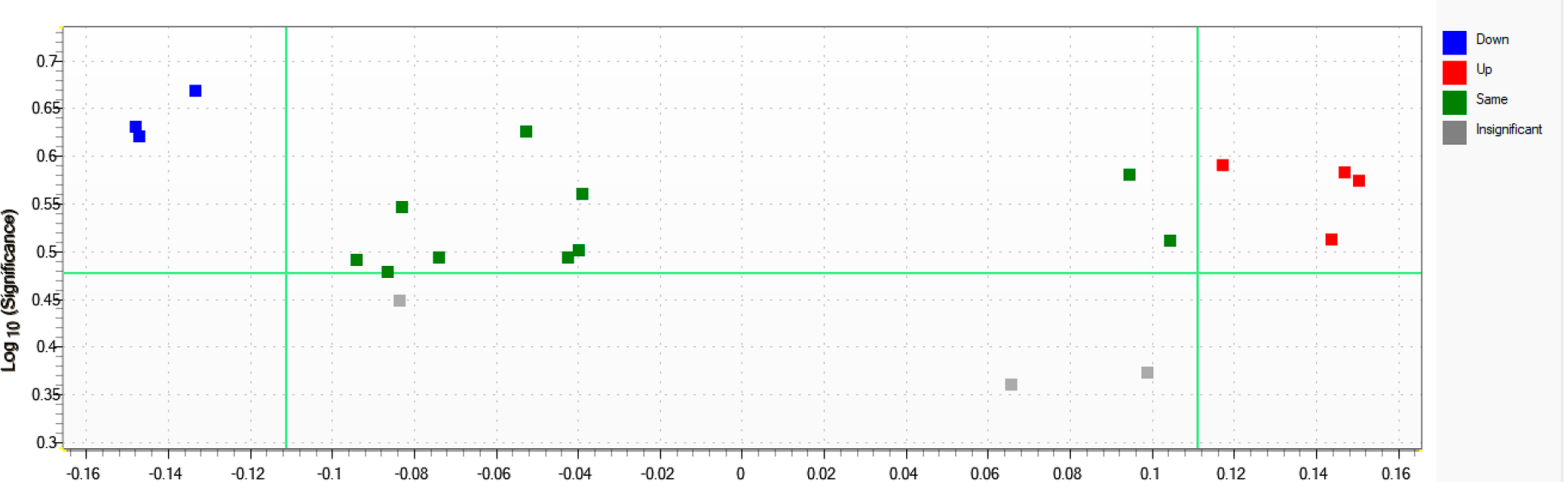
Volcano plot of the tile-based Fisher ratio analysis generated on the GC×GC-TOF-MS data set, indicating cancer-control separation.

A combined approach was applied, utilizing both single-dimensional and multidimensional GC-TOF-MS to maximize biomarker discovery. An assay based on a midpolar column was used for the single-dimensional GC, as the widest coverage choice. Multivariate analysis with OPLS-DA showed separation between the PDAC group and control patients (Figure 3a: R^2^Y, 0.61; Q^2^, 0.32), which was validated from permutation analysis (999 permutations, Figure 3b) and CV-ANOVA with a p-value of 1.4 × 10^–4^. A panel of ten biomarkers was shortlisted based on their VIP scores and biological relevance, presented in Table 1. Furthermore, GC×GC analysis complemented our biomarker discovery efforts, using a polar-based primary column to focus on acidic compounds. Due to the well-known challenges in alignment and coanalysis of large multidimensional chromatographic data sets,^5^ a tile-based approach was utilized. Fisher-ratio-based analysis revealed a further set of seven potentially diagnostic biomarkers (Figure 4), which are presented in Table 2. It is worth noting that three out of four VOCs with potential diagnostic value of our previous study, namely hexanal, 3-hexanone, and p-cymene,^6^ were found in the present work, highlighting the reproducibility and validity of the findings.

**Table 1:** GC-TOF-MS-Derived Candidate VOCs for PDAC Detection

Compound Name	CAS No.	Top Ions (*m*/*z*)	Chemical Class	VIP Score	Levels in PDAC – Fold change	Authentic Standard Confirmation
octanal	124-13-0	100, 110, 84	aldehyde	2.04	↓ −1.25	Yes
hexanal	66-25-1	56, 72, 82	aldehyde	1.87	↓ −1.35	Yes
2-hexenal	6728-26-3	57, 98, 42	aldehyde	1.78	↓ −1.73	-
3-hexanone	589-38-8	57, 71, 43	ketone	1.76	↓ −1.70	-
3-ethylphenol	90-00-6	107, 122, 77	aromatic	1.69	↓ −1.28	-
methyl sorbate	689-89-4	111, 126, 95	ester	1.59	↓ −1.44	-
2-methyl-propanal	78-84-2	72, 43, 41	aldehyde	1.53	↓ −1.37	-
3-methyl-2-butenal	107-86-8	84, 83, 55	aldehyde	1.41	↓ −1.24	-
1-nonen-3-ol	21964-44-3	57, 72, 85	alcohol	1.37	↓ −1.75	Yes
*trans*-2-nonen-1-ol	31502-14-4	57, 41, 82	alcohol	1.36	↓ −1.75	Yes

**Table 2:** GC×GC-TOF-MS-Derived Candidate VOCs for PDAC Detection

Compound Name	CAS No.	Top Ions (*m*/*z*)	Chemical Class	Significance	Levels in PDAC	Authentic Standard Confirmation
p-cresol	106-44-5	107, 108, 93	aromatic	4.67	↓	-
carveol	99-48-9	119, 134, 91	alcohol	4.28	↓	-
2-butylbenzimidazole	5851-44-5	132, 145, 174	aromatic	4.18	↓	-
tetradecane	629-59-4	57, 43, 71	alkane	3.90	↑	Yes
2-methylfuran	534-22-5	82, 81, 53	ether	3.83	↑	-
methyl 2-hydroxy benzoate	119-36-8	120, 92, 52	aromatic	3.75	↑	-
p-cymene	99-87-6	119, 134, 91	aromatic	3.26	↓	Yes

None of the identified VOCs have previously been linked to pancreatic cancer in the literature, except in our previous study.^6^ The majority of the identified biomarkers in the present study are aldehydes, aromatic compounds, and alcohols.

Hexanal has previously been reported in prostate,^11^ bladder,^12^ and lung cancer.^13^ As a short chain aldehyde, it can be produced by peroxidation of unsaturated fatty acids in many parts of the body^14^ and also by oxidation of 2,2,6-trimethyl-cyclohexanone and 3-hexanone.^15^ Urinary 3-hexanone is associated with lung, breast, and colon cancer.^16^ Octanal has been found to be related to clear cell renal cell carcinoma (ccRCC)^17^ and breast cancer.^18^ 2-Hexenal has previously been reported as a biomarker for lung cancer^19^ and head and neck squamous cell carcinoma.^20^ The significantly altered urinary levels of these aldehydes can be a result of altered lipid peroxidation, which resulted in lower levels of the identified aldehydes and has been related with cancer.^21^

p-Cymene has been associated with colorectal cancer, lymphoma, leukemia, and breast cancer.^22,23^ Another aromatic compound, urinary p-cresol, is widely reported to be imbalanced in multiple pathologies and has previously been linked to ccRCC,^17^ lung,^24^ breast, and colon cancer,^25^ leukemia, and lymphoma,^23^ as well as autism spectrum disorder.^26^ P-Cresol participates in a number of metabolic pathways including toluene degradation, nitrotoluene degradation, degradation of aromatic compounds, and protein digestion and absorption,^27^ suggesting its important role in pathophysiological processes and as a more general disease biomarker. Another aromatic compound, 3-ethylphenol, might be further metabolized to ring-dehydroxylated metabolites that lead to oxidative damage, according to Midorikawa et al.^28^

Previous studies have also linked tetradecane with renal cell carcinoma,^29^ asthma, ulcerative colitis, NAFLD, Crohn’s disease, and Celiac disease; and 2-methylfuran with breast and colon cancer,^25^ as well as NAFLD.

## Conclusions

This study has presented an improved tool for global urinary volatolomics. Volatolomic discovery was enhanced in terms of both measured and significant VOCs. This was achieved by employing multiphase sorbent materials, a combination of single-dimensional and multidimensional GC-TOF-MS, and a pragmatic approach in GC×GC data analysis. At the same time, the required urine volume was decreased 5-fold. The applicability of the methodology was showcased in a PDAC cohort, by both confirming previous findings and discovering a series of VOCs with diagnostic potential.
